# Non-linear photochemical pathways in laser-induced atmospheric aerosol formation

**DOI:** 10.1038/srep14978

**Published:** 2015-10-09

**Authors:** Denis Mongin, Jay G. Slowik, Elise Schubert, Jean-Gabriel Brisset, Nicolas Berti, Michel Moret, André S. H. Prévôt, Urs Baltensperger, Jérôme Kasparian, Jean-Pierre Wolf

**Affiliations:** 1 Université de Genève, GAP-Biophotonics, Chemin de Pinchat 22, CH-1211 Geneva 4, Switzerland; 2 Paul Scherrer Institute, Laboratory of Atmospheric Chemistry, CH-5232 Villigen, Switzerland; 3 Max-Born-Institut, Max-Born-Strasse 2A, 12489 Berlin, Germany; 4 Université de Genève, GAP-Non-linear, Chemin de Pinchat 22, CH-1211 Geneva 4, Switzerland

## Abstract

We measured the chemical composition and the size distribution of aerosols generated by femtosecond-Terawatt laser pulses in the atmosphere using an aerosol mass spectrometer (AMS). We show that nitric acid condenses in the form of ammonium nitrate, and that oxidized volatile organics also contribute to particle growth. These two components account for two thirds and one third, respectively, of the dry laser-condensed mass. They appear in two different modes centred at 380 nm and 150 nm. The number concentration of particles between 25 and 300 nm increases by a factor of 15. Pre-existing water droplets strongly increase the oxidative properties of the laser-activated atmosphere, substantially enhancing the condensation of organics under laser illumination.

Self guided filamentation^1^,^2^,^3^,^4^,^5^ of high-power femtosecond laser pulses results from a dynamic balance between the Kerr effect which tends to self-focus the laser beam on one side, and the plasma created by the strong electric field, as well as higher-order polarisation saturation effects^6^,^7^ on the other. The resulting filaments can propagate beyond 100 m in the atmosphere^8^, be created remotely^9^, and withstand adverse atmospheric conditions like clouds^10^ or turbulence^11^,^12^. This non-linear propagation regime is of particular interest for atmospheric applications^13^ such as remote sensing^14^, air lasing^15^,^16^, triggering of high voltage discharges^17^,^18^,^19^ and lightning control^20^. The most striking effect of laser filaments in the atmosphere is laser-induced condensation^21^,^22^,^23^,^24^,^25^,^26^, where the plasma photochemistry induced by laser filaments leads to nucleation of new particles^27^ and their subsequent growth^22^,^28^.

Both laboratory and field studies have shown that laser-induced condensation is associated with the production of locally high ozone and NO_*x*_ concentrations in the gas phase. Furthermore, ion chromatography of laser-produced particles sampled on filters has shown substantial amounts of 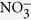 ions in the laser-condensed particles^22^,^29^. The formation of highly hygroscopic HNO_3_ has therefore been identified as one major pathway to support laser-induced condensation of aerosol mass, for relative humidity (RH) in atmosphere higher than 70%^22^. Moreover, ppb-range traces of atmospheric SO_2_ have also been shown to favour laser-induced nucleation of new particles, increasing the number concentration by typically one order of magnitude but without significant total aerosol mass increase. Conversely, laboratory experiments in a cloud chamber showed that ppb-range concentrations of volatile organic compounds (VOCs) lead to an increase in the concentration of laser-induced particles for sizes up to some hundreds nanometers, together with an increase of the condensed mass^27^. This suggests that they both contribute at least to the initial phases of the nucleation but that mostly organic compounds condense on bigger particles. However, very little was known to date about the actual composition of the laser-induced aerosol mass, which is needed to identify the physico-chemical pathways.

Here, we present a detailed chemical characterization of laser-induced aerosols in the real atmosphere. An aerosol mass spectrometer is used to investigate the influence of laser filamentation on ambient particle composition and size distributions. We show that condensation indeed occurs in the atmosphere under illumination by laser filaments, leading to an increase of the total aerosol mass and the appearance of a new size mode. Furthermore, we demonstrate the key contributions of ammonium nitrate (rather than nitric acid, as previously expected), and to a lesser extent, of organics, to the laser-condensed mass. Finally, we show that continuously spraying pre-existing water droplets into the laser beam strongly increases the oxidative properties of the laser-activated atmosphere, substantially enhancing the condensation of organics under laser illumination.

The experimental setup is sketched in Fig. 1. The Teramobile^29^ Ti:sapphire laser delivers Fourier-limited, 180 mJ and 80 fs pulses centered at 800 nm at a repetition rate of 10 Hz. The pulses are loosely focused by an *f* = 25 m telescope into the atmosphere. At the focus, the beam has a waist of around 5 mm (HWHM) and contains ~30 filaments of about 100 μm in diameter each. The focal region is shielded from the wind by an open, galvanized iron tube of 30 cm diameter.

The atmosphere is monitored in the filamenting region at 1 cm side distance from the laser beam by an Aerodyne high-resolution time-of-flight aerosol mass spectrometer (AMS), an optical aerosol sizer, a nanoparticle sensor, and an ozone detector (see Materials and Methods). Additionally, temperature and RH were continuously monitored at the sampling location of these instruments. Unless otherwise specified, the relative humidity was between 80% and 100% during the measurements.

Each experiment consists of a series of 2 to 5 cycles of reference (laser off) and active (laser on) periods of about 15 minutes. The shielding tube is flushed with fresh air from the open atmosphere before each laser-on time period. Besides the nominal conditions described above, we investigated the effect of spraying water droplets in the experimental region using a nebuliser.

We aim at comparing the composition of the pre-existing atmospheric aerosols with those formed under the influence of the laser filaments. We therefore focused on steady-state situations, by averaging the measured size distributions and species concentrations over the active and reference periods. We corrected the measurement baseline for the slow drift of temperature and RH during the experiments. To avoid interference from the transient behaviours of both the instruments and the atmosphere just after starting or stopping the laser, we discarded the time interval of Δ*t* = 4 min at the beginning of each period. This ensures that the average over the remaining time period is stable within 3 standard deviations of the mean. When needed, the effect of the laser was assessed by performing a Student test for the comparison of results from the active and reference periods. Significance is considered achieved for confidence levels of 99% or beyond (*α* ≤ 0.01).

## Results

### Aerosol mass increase

**Figure 1:**
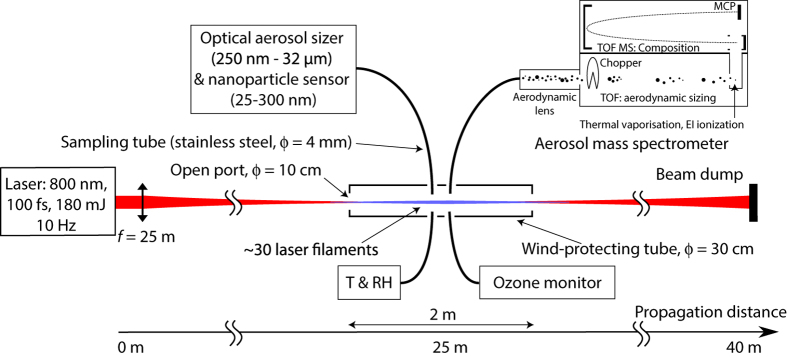
Experimental setup. TOF: Time of flight; MS: Mass spectrometer; EI: Electron impact; MCP: Micro-channel plate.

**Figure 2:**
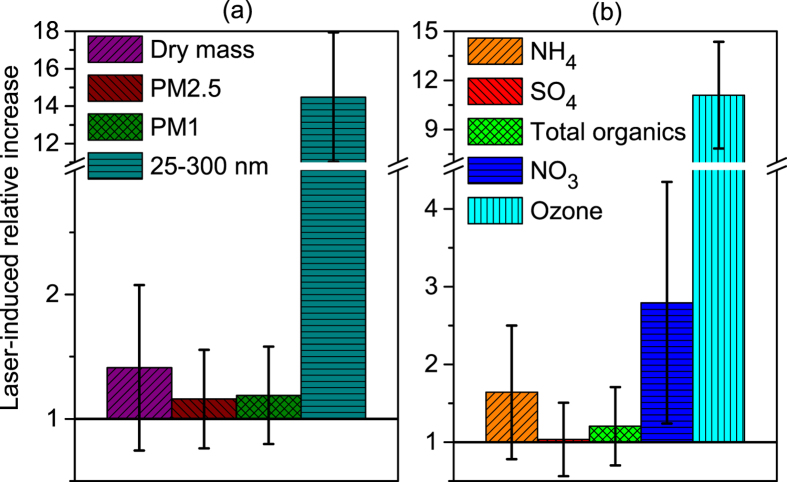
Laser-induced relative increase of (a) the aerosol dry mass concentration measured by the AMS (sum of sulfate, nitrate, organics, ammonium and chloride for vacuum aerodynamic diameters between 60 and 600 nm), the PM1 and PM2.5 mass concentration measured by the optical sizer and the number concentration of 25 to 300 nnm particles detected by the nanoparticle sensor, and (b) the mass concentration of several condensable components detected by the AMS, as well as atmospheric ozone concentration. Error bars correspond to the sum of the relative standard deviations of the values measured with and without laser.

**Figure 3:**
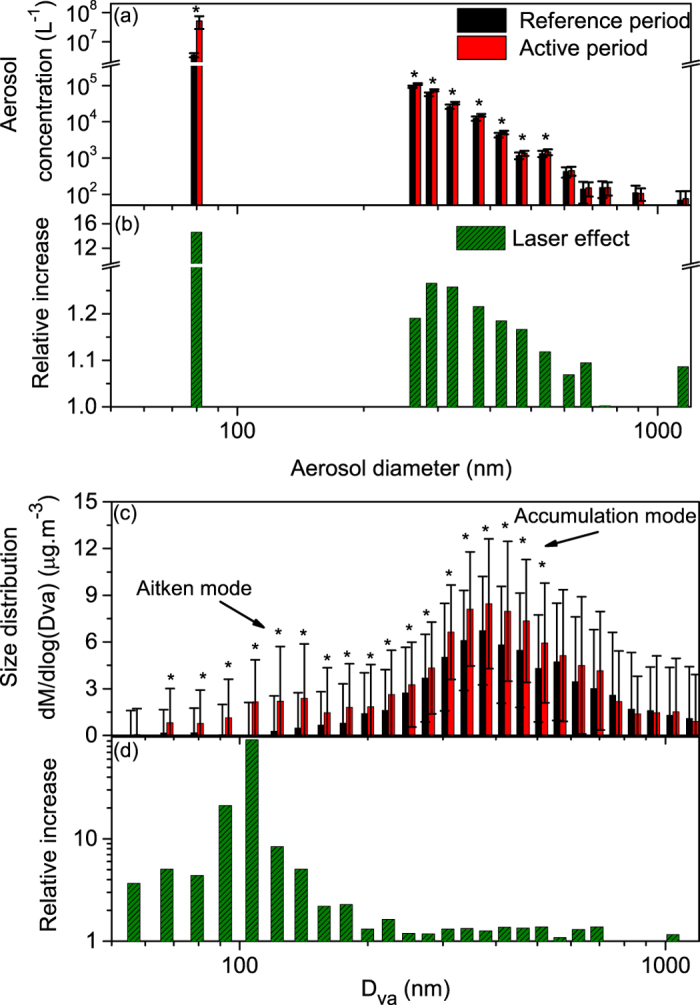
Evolution of the aerosol size distribution under laser illumination. (**a**) Aerosol size distribution between 250 and 900 nm during both reference and active periods measured by the Grimm optical particle sizer, and concentration of 25–300 nm particles measured by the Nanocheck (column centered at the mean diameter 80 nm); (**b**) Corresponding relative increase induced by the laser; (**c**) Dry particle size distribution (total mass detected by the AMS, calculated as the sum of nitrate, sulfate, organics, ammonium and chloride) during both reference and active periods and (**d**) Corresponding relative increase induced by the laser. *D*_va_ is the vacuum aerodynamic diameter. Stars denote size classes where the effect of the laser is statistically significant (*α* ≤ 0.01, see text for details). Error bars correspond to one standard deviation.

The presence of the laser filaments results in a clear increase of the total aerosol mass concentration. As displayed in Fig. 2(a), this increase reaches a factor of 14 in the case of the 25–300 nm particles (~80 nm median diameter). Simultaneously, the concentration of particulate matter with an optical diameter of 250 nm to 1 μm or 2.5 μm (PM1 and PM2.5, respectively) increases by almost 20%, while the AMS measures a 40% increase of the total dry mass (sum of sulfate, nitrate, organics, ammonium and chloride) for aerodynamic diameters between 60 and 600 nm. Such values are in line with previous measurements in the atmosphere^22^. This rise is quite evenly spread over the size classes ranging from 300 to 600 nm, with a statistically significant increase of up to 25% for 300 nm particles (Fig. 3(a)). The large error bars in Figs 2 and 3(c) are primarily due to fluctuations of the laser pulse energy (up to 20%, amplified by the non-linear propagation), the fluctuations of the atmosphere around the laser during the measurements, as well as air currents in the wind-protecting tube (See Fig. 1), which alter the efficiency with which laser-generated mass is sampled. Despite these, the results retain statistical significance. Note however that the error bars on the ratios are calculated as a worst-case scenario by adding the relative errors on each term, providing an upper limit to them.

### Size distribution

**Figure 4:**
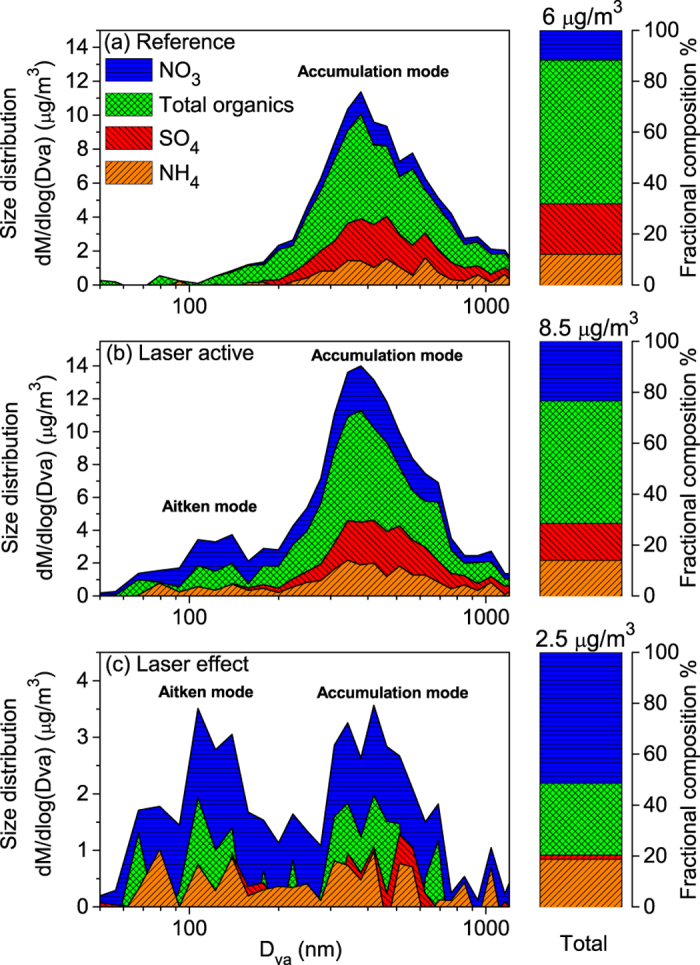
Mass distribution of the measured condensable species within the size distribution during (a) the reference period and (b) the laser active period. (**c**) Difference between the active and reference periods, displaying the condensation effect of the laser. *D*_va_ is the vacuum aerodynamic diameter.

By looking at the total mass detected by the AMS (sum of the nitrate, sulfate, organics, ammonium and chloride), one can see the appearance of a new mode (Aitken mode) centered around 150 nm in the dry particle size distribution when the laser is turned on (Fig. 3(c,d)). This mode could be formed either by nucleation or by growth of nanometric pre-existing particles. Simultaneously, the accumulation mode centered at 380 nm increases by 25%, as also observed in the total mass size distribution. In spite of their different particle sizes, both modes contribute about half of the total laser-induced mass condensation (Fig. 4(c)).

### Chemical composition of laser-induced aerosols

Beyond the total particle mass and number concentrations, the aerosol mass spectrometer allows identifying and quantifying the main components that substantially contribute to the laser-induced condensation. The condensed mass of 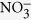 increases by a factor of 2.8, 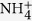 by 60%, and organics by 20% (Fig. 2(b)). Considering their original mass fraction in the aerosols (Fig. 4(a), right column), these components contribute 50%, 20%, and 28% of the net laser-induced increase of the dry mass (Fig. 4(c), right column). These contributions to the laser-condensed mass are comparable in both size modes.

In contrast, no evidence for significant laser-induced 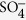 condensation is observed, consistent with the concentration of SO_2_ in the atmosphere during the experiment of 2 μg/m^3^ (as measured by the Geneva city air quality monitoring network^30^).

### Oxygenation of the organic fraction of the aerosols

The AMS allows detailed analysis of the organic aerosol composition. More specifically, oxygenated organic aerosol (OOA) can be described as highly oxygenated, low-volatility (LV-OOA), or less oxygenated semi-volatile OOA (SV-OOA)^32^,^33^. Considering the mass to charge (*m*/*z*) values of 44 (corresponding in particular to 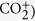 and 43 (mainly C_2_H_3_O^+^), as representative of these two categories respectively, we investigated their relative contributions, hereafter denoted *f*44 and *f*43, to the total organic mass condensed in the aerosols.

**Figure 5:**
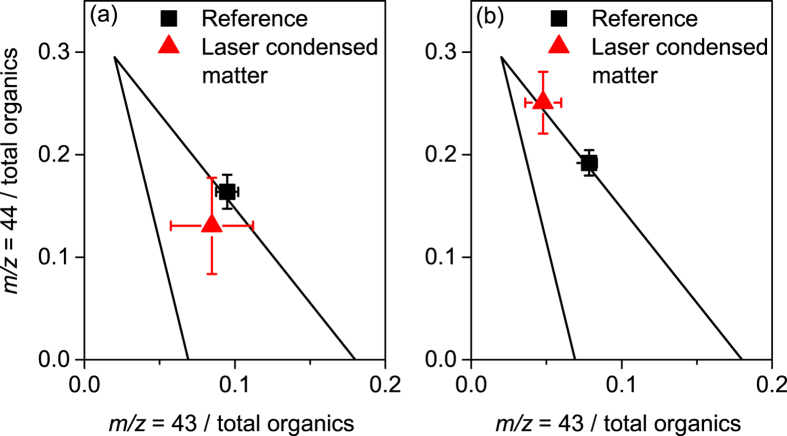
Fraction of highly (*m*/*z* = 44, mainly 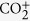) and less oxygenated (*m*/*z* = 43, mainly C_2_H_3_O^+^) organics among the total organics, in particles detected during the reference period (black) and the laser-condensed particles, that is the difference of measurement with and without the laser (red), for (a) the main experiment and (b) when spraying water in a low RH atmosphere (b). The two black lines encompass the region where most atmospheric data are found to date^31^,^32^.

During the experiment, the laser condensed mass presents an *f*44 and *f*43 respectively 25% and 11% lower than in the pre-existing atmospheric aerosol (Fig. 5(a)). This oxygenation state of the laser-condensed organic mass is similar to typical ambient OOA^31^,^32^.

The laser produces highly oxidative conditions, with the generation of local concentrations of O_3_ up to the 100 ppm range^33^,^34^ (see also Fig. 2) and ppbs of ^•^OH radicals in the filaments^35^. These conditions produce OOA with a composition on the boundary of LV-OOA and SV-OOA from the gaseous organics within a few seconds.

### Spraying water droplets to a low RH atmosphere

In an atmosphere with 50% RH, the laser doesn’t condense any mass, and the ambient aerosol is mainly composed of organics (68%), SO_4_ (19%), NH_4_ (8%) and a small percentage of NO_3_ (5%). Continuously spraying water particles close to the laser focus caused the laser to condense mass (as evidenced by a mass increase of 8%), mainly composed of organics (85%). Furthermore, the oxygenation of the laser-condensed organics is higher than the pre-existing ones in this case, as evidenced by the increase of the highly oxygenated organics (see *f*44 in Fig. 5(b)) while the less oxygenated organics (*f*43) simultaneously decrease. The huge amount of ^•^OH produced by the interaction of the water droplets with the laser filaments results in the production of LV-OOA, much more oxygenated than in the main experiment. Conversely, no condensation of hygroscopic species occurs despite the huge amount of water sprayed, indicating that the latter is not available for laser-induced condensation.

### Influence of laser pulse energy

We investigated the effect of the laser pulse energy on the efficiency of laser-induced condensation. Reducing the laser pulse energy from 180 mJ to 110 mJ did not visibly alter the effect of the laser on ambient aerosol. In contrast, at 55 mJ per pulse no effect on the ambient aerosol (i.e. no production of aerosol mass) was observed.

## Discussion

### Laser-induced condensation

The formation of a new Aitken mode accounting for 50% of the laser-condensed dry mass together with the increase of the pre-existing mode provides a clear evidence of the condensation induced by the laser filaments in the atmosphere, an effect that had only been characterised before in laboratory conditions^27^. The formation of new particles by nucleation or growth of pre-existing nanometric particles, as opposed to shattering of larger particles, is confirmed by the increase of the total mass of aerosol detected during the experiment, by the change of chemical composition of their dry mass, and by the lower oxygenation level of the organics contained in the particles detected under laser illumination.

### Condensation of ammonium nitrate

The concentration of NO_3_ and NH_4_ observed in the laser-induced aerosols (50% and almost 20% of the dry condensed mass, respectively, i.e., a total contribution close to 70%) sheds new light on the previously inferred binary water-HNO_3_ condensation under laser illumination. The 0.31 mass ratio of NH_4_ to NO_3_ in the dry condensed mass (Fig. 4), in both the nucleation mode and accumulation modes, is consistent with that of NH_4_NO_3_. One can therefore expect that the hygroscopic HNO_3_ previously identified to assist the condensation of water^22^,^28^ is not condensed, as initially expected, as nitric acid in a binary mixture with water, but rather as hygroscopic ammonium nitrate through the reaction^36^


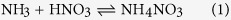


NH_3_ is typically available as background trace gas in the summer sub-urban atmosphere of the experiment location in amounts of several μg/m^3^ 37. This concentration is comparable with the laser-condensed mass of 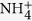, suggesting that the laser-induced condensation relies on the condensation of pre-existing ammonia together with HNO_3_. The latter is generated via the interaction of NO_*x*_ produced by the multi-photon ionisation of N_2_ with O_3_^33^ or ^•^OH radicals^35^, both produced by the multiphoton absorption and photolysis of O_2_. The very low efflorescence of ammonium nitrate and the high quantities of NH_4_NO_3_ in the laser condensed mass suggest that there is always water in the laser induced aerosols. Besides, it validates a posteriori the *ad hoc* introduction of ammonium nitrate for modelling the growth of laser-induced particles^28^.

### Organics

Besides ammonium nitrate, the laser mainly condenses organics (28% of the dry mass). The amount of organics condensed by the laser seems to be driven by its ability to create an oxidative atmosphere. At normal atmospheric conditions and when RH is above 70%, the laser partly oxidises organics, resulting in the condensation of typical ambient LV/SV-OOA, slightly less oxygenated than the pre-existing organic aerosol during our experiment. This suggests that new aerosol mass is formed by the laser from the semi-volatile organics available in the gas phase, and not from the already condensed non-volatile species. The absolute amount slightly below 1 μg/m^3^ of organics condensed by the laser constitutes a few percent of the total concentration of VOCs available in a typical urban atmosphere^38^, confirming the VOC availability for laser-induced oxidation and condensation.

When water is sprayed around the laser filaments at an original RH lower than 70%, the proportion of organics in the dry mass drastically increases (80% of the dry mass). Given that no relative humidity change was observed when spraying water during this particular experiment, we attribute this effect to the interaction of the water droplets sprayed into the laser beam, with the laser filaments. Considering their high concentration during the experiment, about 100 water droplets interact with the laser filaments for each laser pulse. These water droplets then internally focus the incident laser light on a nanometric hot spot, where the extreme incident intensity (in the 10^15^ TW/cm^2^ range) will efficiently ionize water^39^. This ionization produces a concentration of ^•^OH radicals much higher than in normal atmospheric conditions, leading to efficient oxidization of the available organics. The resulting highly oxygenated, low volatility organics then efficiently condense onto particles with a high oxygenation state comparable to that of *α*-pinene particles after exposure to 500 ppb of O_3_ during 13 hours^31^,^32^,^40^. This very fast oxidization illustrates again the extreme conditions locally produced by the laser filaments.

Our results evidence the dual role of organics in laser-induced condensation. While they had been shown to promote nucleation in a background-free atmosphere^27^, we show that they also substantially contribute to the particle growth up to at least 600 nm in diameter (see Fig. 4).

### Sulfate

Sulfuric acid is known to provide one of the main sources of condensation nuclei in the atmsophere^36^. In the case of laser-induced nucleation, experiments in a cloud chamber showed that sulfuric acid does not only drastically enhance the number density of nucleated particles, but also the total condensed mass^27^. The very low mass fraction of sulfate ions in the laser-induced condensed mass (<2%) detected during our experiment can be explained by the low ambient SO_2_ concentrations during the study. In this case, its role is restricted to the formation of small condensation nuclei, most probably together with oxidized organics^41^.

### Laser energy

The energy dependence of the efficiency of laser-induced condensation appears to display a threshold between 50 and 110 mJ. This suggests that the effect of the laser can be understood by considering the interplay between two phenomena.

On one side, the efficiency of the plasma photochemistry increases non-linearly with increasing incident laser pulse intensity. At 800 nm, the photolysis of O_2_, its ionization, and that of N_2_ respectively require 4, 8, and 11 photons^3^. The associated efficiencies therefore depend on the corresponding powers of the laser input intensity, so that the condensation mechanisms initiated by these processes will successively come into play when the laser energy rises. This results in the observed dependence with the pulse energy. On the other side, however, particle formation and growth requires the availability of condensable species in the volume swept by the high intensity laser, or at reach within a reasonable transport time. As this transport mainly occurs by diffusion, which is very slow for distances beyond the mm-range, diffusion constitutes the limiting factor to condensation when the laser pulse energy is further increased.

This finding illustrates that increasing the magnitude of the laser effect requires to increase the activated volume, by either increasing the beam diameter, or by steering the beam dynamically.

## Conclusion

We simultaneously measured the size distribution and the quantitative size resolved aerosol composition by mass spectrometry during laser-induced condensation in the real atmosphere. We obtained direct evidence of laser-assisted condensation in real atmosphere, with a 40% increase of aerosol total mass and a 15-fold increase in the particle number concentration between 25 and 300 nm. Furthermore, we observed the condensation of hygroscopic NH_4_NO_3_ accounting for two thirds of the laser-condensed mass. This abundance shows that NO_3_ condenses as ammonium nitrate, rather than as nitric acid as previously expected. The rest of the laser-condensed dry mass is mainly provided by organics, accounting for as much as 28%, evidencing their role in the particle growth. The presence of these two components (ammonium nitrate and organics) can be attributed to two main condensation mechanisms related to the production of nitrogen oxides, ozone and ^•^OH radicals by the interaction of the filaments with the atmosphere. The first one consists of the generation of HNO_3_, which then condenses in a ternary system together with NH_3_ and water to form NH_4_NO_3_ in the laser-induced particles. The second one is the oxidation of volatile organics by ^•^OH radicals and O_3_ into less volatile organics which condense on the pre-existing particles. Moreover, the oxidation as well as the degree of oxygenation of organics can be strongly increased by the presence of pre-existing water droplets because of increased ^•^OH radical concentrations due to internal focusing of the laser light within the droplets. Our work therefore offers a global picture of the complex mechanisms at play in laser-induced condensation.

## Methods

### Teramobile laser

The Teramobile^30^ is a Ti:sapphire laser delivering 80 fs pulses of 180 mJ, at a repetition rate of 10 Hz with a wavelength centered at 800 nm. The beam is sent horizontally into the open atmosphere, 120 cm above ground. It has an initial diameter of 7.5 cm and is loosely focused by an *f* = 25 m telescope. The beam at the focus has a waist of around 5 mm (HWHM) and contains ~30 filaments. The beam is blocked by a screen after a further 15 m of propagation to avoid any interference by particles ablated from this beam dump.

### Wind-protecting tube

The region around the focus is shielded from wind by a 2 m long, 30 cm diameter galvanized iron tube. It is open at each end, with 10 cm diameter ports letting both the laser beam get through and exchanges with the free atmosphere to occur. The detection instruments continuously monitored the atmosphere at 1 cm distance from the laser beam close to the filaments, via 1.5 m-long, 4 mm inner diameter copper sampling tubes connected to the center of this shielding tube.

### AMS

Size-resolved non-refractory particle composition was measured using an Aerodyne high-resolution time-of-flight aerosol mass spectrometer (AMS)^42^,^43^. Briefly, the AMS continuously samples particles from ambient air (0.8 L/min) through a 100 μm critical orifice into an aerodynamic lens (2 torr). Here the particles are focused into a narrow beam and accelerated to a velocity that is inversely related to their vacuum aerodynamic diameter. The particle beam can be either alternately blocked and unblocked (yielding ensemble mass spectra of the incident aerosol), or modulated by a spinning chopper wheel (150 Hz), yielding size-resolved mass spectra at the cost of lower sensitivity. The particle ionisation on a resistively heated surface (600 °C, 10^−7^ torr), where the non-refractory components flash vaporize, are ionized by electron impact (70 eV) and are detected by time-of-flight mass spectrometry. The AMS can detect most atmospherically-relevant species except for black carbon and mineral dust, while water is not quantitatively detected due to high instrument backgrounds and uncertainty over the fraction of water that evaporates in the aerodynamic lens. The lens has unit transmission efficiency for particles with vacuum aerodynamic diameters between 100 and 600 nm, and approximately 10% transmission of 50 and 1000 nm particles^44^. Mass spectra are analyzed and quantitatively split into e.g. nitrate, sulfate, ammonium, and organics using the standard fragmentation table-based analysis of Allan *et al.*^45^. Depending on the experiment, the AMS was operated with either 1 min or 20 sec time resolution.

### Particle sizer

The aerosol sizer is a Grimm 1.109 measuring the aerosol size distribution by optical scattering in 31 classes ranging from 250 nm to 30 μm. This instrument samples 1.5 L/min and provides measurement at a temporal resolution of 6 s, although the overall reaction time of the instrument and the associated sampling tube can be estimated to lie in the 8 s range. It also integrates the size distribution up to 1 and 2.5 μm diameter, respectively, to estimate the PM1 and PM2.5 mass concentration in air.

### Nanoparticle sensor

A nanoparticle sensor (Grimm Nanocheck 1.365) measures the 25–300 nm particle number density. Sampling flow and temporal resolution are the same as the above particle sizer.

### Ozone detector

The ozone detector is a Horiba APOA-350E and sampled the atmosphere at a rate of 2 L/min, measuring ozone concentration with a temporal resolution of 20 s.

### Temperature and RH probe

The device monitoring temperature and RH is a Testo 635-2 device. Its probe is located at the same place as the other instruments.

### Nebuliser

The nebuliser used to spray water is a TSI 3076 nebuliser equipped with a 3074B air filter. The corresponding size distribution was centred around 1.5 μm, with a geometric standard deviation smaller than 2.

## Additional Information

**How to cite this article**: Mongin, D. *et al.* Non-linear photochemical pathways in laser-induced atmospheric aerosol formation. *Sci. Rep.* **5**, 14978; doi: 10.1038/srep14978 (2015).
